# Current status and future prospects for enabling chemistry technology in the drug discovery process

**DOI:** 10.12688/f1000research.9515.1

**Published:** 2016-09-30

**Authors:** Stevan W. Djuric, Charles W. Hutchins, Nari N. Talaty

**Affiliations:** 1 Discovery Chemistry and Technology, AbbVie, North Chicago, IL, USA

**Keywords:** Chemistry technologies, drug discovery, parallel synthesis chemistry, photochemistry, electrochemistry, computer-assisted drug design

## Abstract

This review covers recent advances in the implementation of enabling chemistry technologies into the drug discovery process. Areas covered include parallel synthesis chemistry, high-throughput experimentation, automated synthesis and purification methods, flow chemistry methodology including photochemistry, electrochemistry, and the handling of “dangerous” reagents. Also featured are advances in the “computer-assisted drug design” area and the expanding application of novel mass spectrometry-based techniques to a wide range of drug discovery activities.

## Introduction

Recently, Paul *et al.* ^1^, among others, have reiterated that the pharmaceutical industry has “to substantially increase the number and quality of innovative, cost-effective new medicines, without incurring unsustainable R&D costs”. We feel that chemistry technology initiatives can provide significant value in the areas of cycle time reduction, cost of goods, and probability of success. Our efforts in this area have evolved over several years but have invariably been aligned with a principle propounded by George Whitesides, namely that “you don’t really know you have solved the problem for someone until they like your solution so much they’re willing to pay you to use it” ^2^. For a platform chemistry technology organization, this means that important innovations are those that deliver robust, long-term impact on projects and that internal scientists view as indispensable/significant assets.

Such an organization, like AbbVie, may comprise multiple units including centralized platforms such as high-throughput chemistry/parallel synthesis ^3^, DNA encoded libraries ^4– 6^ (either internally or through collaboration), new synthetic chemistry technology development (including photochemistry ^7– 14^, electrochemistry ^15– 19^, high temperature chemistry ^20– 22^, “hazardous” chemistry ^23, 24^, flash chemistry ^25^, multiple component reactions [MCRs] ^26, 27^, etc.), integrated synthesis/purification/bioassay systems ^28, 29^ plus analytical chemistry technologies such as high-throughput purification, chiral separations ^30^, and mass-spectrometry-based techniques such as nanodroplet accelerated chemistry, small-molecule mass spectrometric imaging, etc. The organization may also include a computer-assisted drug design (CADD) group and a fragment-based discovery group. Moreover, as of late, increased investments have been made in the chemical biology area, particularly in the area of chemical proteomics ^31– 33^ and intracellular target engagement properties of compounds ^34– 36^. Technologies such as PROTACs have also gained steam over the last few years with several companies either developing internal programs or entering into collaborations ^37^. These latter technologies (and others) being, in part, fueled by the industry’s desperate need to identify and validate quality, new small-molecule targets and to tackle more “difficult-to-drug” targets in the proteome.

## Chemistry technology in the drug discovery process

This review/viewpoint will focus predominantly and briefly on synthetic chemistry technology, CADD, and the application of mass spectrometric techniques to drug discovery operations.

In the parallel synthesis space, we and others have endeavored to speed up turnaround time for the traditional synthesis, purification, analysis, and registration cycle for compound library production, which historically has averaged at between a week and ten days. Integration of synthesis and purification in flow format has now been achieved by several groups with, in our case, a turnaround time of 2–3 days from synthesis to compound registration (using segmented flow techniques) ^38– 40^. Some groups have integrated this paradigm with bioassay and software that enables the next round of compound synthesis to be predicted ^41– 43^. In concert with efforts to reduce cycle time for parallel synthesis and its overall value to an organization, many groups have established programs to accumulate a significant collection (many thousands) of building blocks (BBs), many of which are of a custom nature. These BBs (or monomers/reagents) are either stored in-house or shipped directly from the vendor (who may actually function as a compound repository for the company). Several companies feel that access to proprietary BBs provides a competitive advantage, whilst others argue that novelty is actually time dependent and not worth the investment.

A significant issue for high-throughput chemistry groups is the production of compound libraries (24–96 compounds, in general) in quantities necessary for primary and secondary bioassay. As time does not permit the optimization of all reaction components, yields often (even with well-known chemistries, e.g. Buchwald and Negishi reactions) can be low. To this end, many companies have been evaluating high-throughput experimentation techniques to address some of these problems. For example, chemists at Merck, using a miniaturized, high-throughput automation platform that examines how synthetic molecules react under different conditions, were able to perform more than 1500 chemistry experiments in less than a day. This technique could accelerate the process of drug discovery and allow chemists to more easily study new medicinal compounds ^44^.

An important component of the contemporary high-throughput chemistry laboratory is the incorporation of automation into the workflow, be it automated reaction execution or allied high-throughput purification and analysis of compounds. Many pharmaceutical groups that feature highly automated platforms, e.g. Lilly, BMS, Merck, and AbbVie, have either internal automation engineering groups or alliances with specialist automation vendors.

The use of flow chemistry has undergone explosive growth over the last 10 or so years with the Ley, Jensen, and Kappe groups (amongst many others) being notable for their outstanding contributions ^44– 49^. Moreover, pharmaceutical companies have many significant investments in continuous flow processing capabilities on the plant scale, particularly for the use of hazardous reagents (e.g. nitrations or azide chemistry). Uptake in discovery organizations has been less dramatic (notwithstanding the applications mentioned previously), but certainly flow chemistry has allowed for the routine use of fluorinating reagents and troublesome reagents such as diazomethane on a larger scale ^50, 51^.

Photochemistry has become in vogue over the last number of years for several reasons, including its ability to generate structures of greater molecular complexity/Fsp3 count, e.g. small ring forming capability ^52^, and in the case of photoredox chemistry to expand the synthetic chemistry toolbox ^53^. The advantage of flow-based photochemical protocols over traditional batch processes has been propounded and discussed ^54^. Many examples of novel photoredox reactions have been described in the literature over the last 5 or so years by academic groups, whilst, in the pharmaceutical domain, Merck (amongst others) have described many useful applications of the technology and have invested in large-scale facilities ^55^.

Electrochemically enabled transformations appear to be gaining traction, particularly in the process area. The attractiveness of the “green chemistry” aspect of this technology, especially electrochemical oxidation, is likely a driving force as well as its low cost ^56^. Flow electrochemistry techniques have been described and commercially available reactors are now available ^57^.

It would be remiss not to mention the value of multiple component reaction chemistry (MCR) to the production of libraries of compounds that have made it into high-throughput screening decks and provided leads for many nascent Hit to Lead campaigns. The value of inherent chemical methodology itself that is highly efficient, atom economic, green, and tremendously enabling with regard to rapid follow up of new “hits” is high. This particular field of research, initially driven by individuals in industry and now literally exploding in the academic sector, is often denoted MCR platform technology – tremendously powerful considering the ever-increasing number of new succinct 1–2 step routes to known heterocycles and similarly succinct routes to new chemotypes for which hit generation efforts are in desperate need of as we engage intrinsically new targets that often require ligands occupying regions of chemical space that have been previously unexplored. Many excellent reviews of this burgeoning area have been published over the last several years, including those of the Hulme and Domling groups ^26, 27^.

High temperature chemistry has been utilized by several pharmaceutical and technology-based groups for the synthesis of pharmaceutically relevant heterocycles with the use of high temperature flow reactors featuring prominently ^58– 62^. It is certainly our experience that many sluggish reactions or reactions requiring forcing conditions can be conducted effectively at elevated temperature in a matter of minutes in good yield ^63, 64^. Commercially available systems are now available, including the Thales Phoenix reactor ^65^.

What of the future of chemistry technology? Ley, Baxendale, and others have extensively described machine-enabled synthesis ^66, 67^. Some of this is already in place in the industry context and has demonstrated value (as highlighted above). Will we see the advent of robochemists doing routine chemical reactions including set up and work up in the laboratory in the next 10 or so years (the 8pm to 6am shift) ^68– 71^? Will structure–activity relationship prediction and development be done through artificial intelligence/deep learning approaches with oversight by a select number of experienced medicinal chemists? Certainly, significant challenges need to be overcome before these possibilities can come to fruition; however, efforts are underway to develop these approaches. It will be interesting to see the industry’s openness to these transformational “culture-changing” initiatives in the future.

## Recent advances in computer-assisted drug design

Twenty-five years ago, the practice of CADD was carried out by a few acolytes with the knowledge of physical organic chemistry and the ability to deal with the early, slow command-line driven software of the time. Since then, computers have become faster, software has become much easier to use, and the practice of all aspects of CADD has spread widely to all corners of the pharmaceutical industry and many educational institutions. Here we will touch on just a few of the highlights marking the progress of the disciplines of cheminformatics, molecular modeling, structural biology, and structure-based drug design. Equally impressive examples of CADD have been shown for biologics design and development sciences.

One of the major, recent advances has been in the practice of CADD. While the practice used to be carried out by a few and the results relayed and interpreted to synthetic chemists, now many chemists have become more conversant, more familiar, and even expert in some aspects of CADD. It is now common for chemists to have a detailed knowledge of the three-dimensional landscape of the binding site of the target protein. The practice of CADD has also evolved from merely a quest for binding affinity or altering the function of a system to a search for molecules that could become drugs with proper pharmacokinetics (PK), toxicology, IP, and affinity. CADD has moved far towards *drug* design, not just ligand finding. This is due to software platforms that combine computation and the immediate population of new biological data with the ability to correlate structure properties with any parameter ^72, 73^. It is also due to the new development of predictive models of many PK parameters and some toxicological parameters ^74^. Some of these models are classification or regression-equation based. Many are knowledge based, using the information from compounds that have been tested to predict the data for other close analogs. Knowledge-based models are used in molecular docking, binding affinity prediction, and PK parameter development, among others ^75– 77^.

Clearly, the speed and graphics capabilities of modern computers have been a major asset. But even that can be a limiting factor in virtual ligand screening or molecular dynamics. The accessibility of multi-core computers, local clusters, and calculations on graphics processors (GPUs) and now cloud-based systems where a very large number of CPUs can be tied together on demand has made hardware less of an issue. Problems that could never be thought of as approachable can now be confronted ^78^. Likewise, the accessibility of free software to carry out nearly every aspect of CADD has made the barrier to carry out computations quite low ^79^. Now the major impediment to good science is the human. It is the thinking of which problems to attack with the proper tools and the interpretation of the results and perhaps the redoing of the calculation which is the slow, but the most important, step in CADD.

The traditional realm of CADD has been small-molecule ligand development, and many of the same challenges continue in the transition to drug development. Structural biology has made impressive strides in the ability to obtain crystal structures of protein targets, membrane-bound proteins, and huge complexes ^80^. The use of synchrotrons has revolutionized the speed and quantity of structures solved per year. Some of this has to do with the ability to use smaller crystals, even “invisible” crystals ^81^! The pharmaceutical industry continues to solve many structures for proprietary use, which then sit on the shelf after the project has terminated. Efforts are underway to allow sharing of these structures in the precompetitive space ^82^. One of the major advances in structure solution is the ability to solve the structures of membrane-bound G-protein-coupled receptors. This has dramatically changed the way these targets can now be prosecuted ^83, 84^.

A few techniques or methodologies have been highlighted by their widespread use or recurrent interest. The use of fragment-based methods, the new interest in water and its energetics, and the renewed interest in free-energy perturbation (FEP) are spotlighted. The identification of fragments that bind to a protein of interest and the expansion of that hit into more potent leads has become the “go-to” method to rapidly develop novel high-quality ligands ^85, 86^. The use of multiplexed surface plasmon resonance (SPR) instruments has allowed the rapid screening of fragment libraries using small quantities of label-free protein, even of membrane-bound proteins ^87^. The determination of the binding location and pose of the fragment continues to be done best by X-ray crystallography, although nuclear magnetic resonance spectroscopy (NMR) can also be used. Computational methods to determine viable fragment poses for proteins that are not yet amenable to X-ray crystallography continue to be an area of interest ^88^. Methods to better determine the pose and then suggest what to do next with the fragment, what other fragment libraries should be tested, or how to combine it with other fragments are important future priorities.

Water has begun to be treated seriously as more than just something to fill a binding site. A raft of new methods have surfaced recently ^89– 92^ to calculate, in different ways, the energetics of individual waters, which allows chemists to begin to think of ways to use these data in drug design ^93^. Strategic choices of which waters to replace, which to interact with, and the potential consequences of each strategy can now be evaluated. While the programs produce numbers reflective of the energetics of the waters, it is incumbent on the user to understand the protein site and understand the basis of the values in a more holistic manner.

The interest in FEP has increased recently with easier-to-use software and faster computers taking the calculation out of the realm of the very few who knew what they were doing and now allowing the less experienced to carry out these calculations ^94^. Rather than do just one individual change, one is now able to simultaneously calculate the energetics of changing a collection of molecules into each other. It is safe to say that the new methodology is in the testing phase, especially in the pharmaceutical industry. For these tests, high-quality X-ray structures of the individual molecules in the target protein must be supported by equally high-quality, reliable biological testing. With each pharmaceutical company having only a few data sets that fill these criteria, this presents another opportunity for data sharing ^82^. One of the issues in these FEP calculations is the waters in the binding site and how they may change with different substituents. One of the interesting twists is to combine the FEP of a ligand in a site and the water in the same site by simultaneously, slowly swapping the ligand for an equal-volume group of waters to determine the binding affinity ^95, 96^. While this may be a difficult task, carrying out this calculation for known molecules would allow one to scale results for unknown molecules.

The quest for the ability to predict the binding affinity of new analogs continues ^97– 99^. Many methods have been developed over the years and one might say an unsatisfactory plateau has been reached in prediction ability when comparing R ^2^ and RMSE of experimental versus calculated affinities of tens to hundreds of data points. Is this a consequence of the methods or the data? Do we really understand the biophysics of ligand binding? Are the experimental data perfect? How do we account for experimental error and lack of understanding of all the factors affecting ligand binding ^100^? In the practical use of these results, is that what we really need? Rather, might it be better to do a pairwise comparison where the affinity of compound A is known and the simple question is whether hypothetical molecule B will be better or worse? Rather than aim for the best molecule as regression-based models suggest and be disappointed when the desired outcome is not achieved, might it be better to know with high confidence that the new molecule will be better and therefore approach high affinity in a stepwise manner? This might be the application domain of current methods or super-fast FEP calculations.

The combination of new methods in cheminformatics to develop models of PK parameters, new ability to solve X-ray structures of complex biological systems, and new awareness of water and other factors affecting binding affinity has expanded the use of CADD to *drug* design. Significant challenges remain in the field, including the Holy Grail of predicting whether that next molecule will be a dud or a drug.

## Recent advances in mass spectrometry

Mass spectrometry is an analytical chemistry technique in which chemical species are ionized and then identified and quantified by measuring their mass-to-charge ratio and abundance of gas-phase ions. The fundamental principles of mass spectrometry are over a century old. That it is still growing is a testament to the commitment of the researchers and manufacturers who continue to come up with innovative solutions to address increasingly complicated questions. The next generation of mass spectrometers continues to evolve and is faster and more sensitive, with some models featuring ultrahigh-resolution capability. This makes mass spectrometry one of the most versatile technologies in drug discovery finding applications across the entire pharmaceutical pipeline ^101– 103^. Here, we will not address routine uses for mass spectrometry but will focus on the more innovative, revolutionary, and sometimes controversial techniques within the mass spectrometry community which have yet to garner broader acceptance.

In a drug discovery organization, mass spectrometry routinely provides medicinal chemistry support by determining compound identity and purity. Purity is often co-determined by NMR and robust improved liquid chromatography (LC) detectors such as the CAD, ELSD, UV, and NQADs ^104^. Faster scanning quadrupoles coupled to ultra-performance LC (UPLC) systems with robots and plate feeders provide increased speeds and capacity. Miniature mass spectrometers are increasingly finding their way into fume-hoods as a rapid solution for chemists to quickly confirm the identity of what has been synthesized ^105, 106^. However, in a platform technology organization, the trio of speed, quality, and cost need to be effectively balanced. The mini-mass spectrometers have yet to gain a broader acceptance from the medical chemistry community and analytical chemists.

A new and exciting area into which mass spectrometry is making a foray is in its application as a synthetic and online reaction monitoring platform. With the recent advances in ambient ionization, the ion source is back in focus as an active area of research ^107, 108^. Bimolecular reactions occurring in confined volumes of solution (microdroplets or thin films) can be accelerated by orders of magnitude for simple derivatization and acid/base reactions ^109^. These microdroplets can subsequently be used in reaction monitoring (to identify intermediates, follow kinetics, and deduce mechanisms) and also in chemical synthesis (small-scale preparative mass spectrometry). Synthesis is carried out by electrosonic spray ionization (ESSI) with on-line mass spectrometry followed by deposition on a collector surface ^110, 111^. Multiplexing sprayers will enable scale up, with the 100 mg scale being targeted. To push the limits of this technology, heterogeneous reactions, air- and water-sensitive reactions, and metal-catalyzed coupling reactions are being currently investigated.

With the advent of specially designed ultrafast mass spectrometers targeted for high-throughput screening applications (e.g. time of flight [TOF] analyzers), sampling speeds in the realms of less than one sample per second are now possible. Early this year, Bruker showcased a MALDI-based high-throughput screening instrument, the rapifleX MALDI PharmaPulse, capable of obtaining data at about three samples per second, thereby enabling primary screens by mass spectrometry. The automated sample loading component is currently under development and is being evaluated at GlaxoSmithKline ^112^. The MALDI system also requires the application of a matrix and does not use any sample cleanup. Laser diode thermal desorption (LDTD) is another laser-based high-speed platform currently being offered by Phytronix and being implemented in the pharmaceutical industry. It has sampling speeds of one sample every six seconds. Indirect thermal desorption of sample occurs using a precisely controlled laser diode and ions are then transported to a mass analyzer ^113^. Acoustic mass spectrometry is a new technology which was recently highlighted at the 2016 Society for Laboratory Automation and Screening meeting. In an acoustic mass spectrometry experiment, instead of using a needle to aspirate and spray the samples, a sonic pulse is sent through liquid, rapidly creating spray-like conditions. These events happen at speeds of 500 Hz and can theoretically generate 10,000 data points per hour. Practical scanning speeds approach three samples per second. Efforts are currently underway to implement this platform for high-throughput screening ^114^. The widely accepted and established platform for fast analysis by mass spectrometry is the Agilent rapid-fire system, which allows for sample cleanup using a SPE cartridge, thereby providing capability for high-content assays. Data are routinely acquired at sample speeds of one sample every eight seconds ^115^.

High-end mass analyzers such as the Q-TOFs, TOF-TOFs, Orbitraps, and FTICRs are routinely used in “omics” (metabolomics and proteomics) initiatives. Key applications are directed to understanding disease biology, new target discovery, identifying PK/PD markers, and in imaging applications ^101– 103, 116^. An imaging experiment can help to redefine the “D” in ADME studies by determining the spatial distribution of therapeutics or endogenous molecules in a thin tissue section. Advances in instrumentation, matrix application, quantification, and processing software have enabled several pharmaceutical companies to routinely implement this technology to determine drug/metabolite distribution, target engagement, and confirm and explain toxicology findings ^117– 119^. MALDI is the preferred imaging platform; however, newer techniques such as DESI, MALDESI, LAESI, LESA, and FlowProbe are increasingly being used as they are fitted onto topline mass spectrometers ^120^. DESI has also found use in product protection initiatives (adulteration and counterfeit testing) ^121, 122^.

Two approaches appear to be defining the future of mass spectrometry: coupling previously incompatible techniques together or improving the way mature technologies are coupled to newer mass spectrometers. A few examples of each approach are worthy of mention. Every instrument vendor now has an improved ion-mobility source. Bruker presented the TIMS-TOF (combines ion trapping with ion mobility) ^123^ as one of its highlights at this year’s ASMS meeting. Other major vendors such as Agilent, Sciex, Thermo, and Waters all offer ion mobility on their current instruments. Capillary electrophoresis is now being coupled to multiple instruments using a ZipChip CE-ESI microfluidics paradigm developed by a startup company, 908 Devices ^124^. Atomic force microscopy and mass spectrometry are being combined at Oak Ridge National Labs by Kertezs and colleagues and the startup Anasys for imaging applications at ultrahigh spatial resolutions ^125^. Mass cytometry combines the techniques of flow cytometry and mass spectrometry. This enables the measurement of over 40 simultaneous cellular parameters at a single-cell resolution, which is a significant improvement over fluorescence methods. This technology is available commercially ^126, 127^. Multiplexed ion beam imaging (MIBI) combines immunohistochemistry with mass spectrometry (MSIHC); this allows imaging of up to 50 proteins at super-high spatial resolution ^128^.

With multiple innovations in the near future and startup companies partnering with established companies to design custom solutions, the future of mass spectrometry applied to drug discovery appears to be bright.

## Concluding remarks

Summarily, we have tried in this article to provide a perspective on recent developments in the area of enabling chemistry technology as applied to drug discovery efforts in the pharmaceutical industry. Some of these initiatives are now becoming mainstream, while others are at an early stage. Not all will bear fruit. Practioners in the area must be willing to suffer some failures if they wish to be on the cutting edge of technology development, whether done internally or through external collaboration. In our experience, this is a worthwhile risk if one wants to lead rather than follow.
